# Abstract of Proceedings of Buffalo Medical Association

**Published:** 1874-05

**Authors:** E. R. Barnes

**Affiliations:** Secretary


### ART. II.—*Abstract of Proceedings of Buffalo Medical Association.*

Reported by E. R. Barnes, M. D., Secretary.

At the Annual Meeting of the Buffalo Medical Association, held at its rooms in the Medical College; there were present, the President, Dr. Hauenstein in the chair, and Drs. Rochester, Miner, Gould, Lothrop, Bailey, Nicheil, Briggs, Lynde, Samo, Shaw, Wetmore, Fowler, Brecht, Walsh, Diehl, Strong, Barnes and Harvey.

The minutes of the last meeting were read and approved. The Secretary and the Treasurer respectively presented their reports, which were received and ordered to be placed on file.

The President, Dr. Hauenstein, then made the following valedictory address:

Gentlemen:—In obedience to an established custom, the retiring president, before transferring to his successor, the trust committed to his care when elected, is expected to have something to say; and since he is at liberty to choose his own subject, he is almost debarred from making his exit with a simple excuse.

Conscious of my failings, I hesitate to say what little I intend to say, and would prefer to be silent, had it not the appearance of shirking a duty which the honor of the position requires of me. But I trust that your expectations are modest, for if not, you will be sadly disappointed.

This association was organized nearly twenty-four years ago. In its list of membership are names of men who take an enviable and exalted position among the medical profession of the United States. The association's published proceedings have been perused with avidity for the many practical facts and suggestions therein contained. The interest manifested in its doings were not confined to this city alone, but extracts from its proceedings were published in many of the Medical Journals all over the land. In short it had a vital existence, and its members could point with a feeling of gratification and pride to its record. Such has been the condition of this association until of late.

By-removal to other cities and by death, we regret and mourn the loss of most eminent abilities, men distinguished in the different departments of our profession, and whose contributions to this association stamped it with an interest and importance second to but few of its kind.

To-day, however great as has been the loss I have indicated, the talent represented by the members of this association, is such as will compare most favorably with the talent of kindred organizations in other parts of the country. To-day, yet more interest attaches to the progress of medical science than in years passed, and the importance and usefulness of institutions like ours, remains an acknowledged fact. But have we any reason to congratulate ourselves on the flourishing condition of the association? Will the record of its doings for the passed year or two, vindicate it against the charge that its vitality is impaired? Are we stronger, numerically? Have its regular meetings been attended in numbers to indicate an interest for its prosperity? How gladly would I answer these questions in the affirmative, but truth and candor say no!

It follows that a review of the doings of this Association for the past year would neither be gratifying nor instructive, I therefore leave the task, but with a feeling of regret, that in a profession so numerous as ours in this city is, so little interest should be manifested for an institution, having the welfare of all in view. What is the cause, a physician will almost instinctively ask, yet no one knows as well as he, how unsatisfactory the answer to such question generally is. The subject of cause to effect is one upon which more words are wasted than upon any other subject, a verification of which I fear might result, if I were to answer the present question, and even were I successful in its solution, in conformity with truth, it would amount to but a thankless task when performed. I will therefore not attempt an examination of what to me seem causes which disturb the *esprit de corps* and paralyze the usefulness of this Association. But this much I will say, *we cannot all be Professors.*

Happily, we find that the anatomy of a professor bears a striking resemblance to that of other mortals, and that the same laws govern all in common. To be a competent Teacher of Medicine, is certainly a position of honor and great usefulness, demanding our respect. But it follows not that all honor and merit are associated, exclusively, with that title, and however anxious we may be to aspire to that eminence, a disappointment is no dishonor.

Whence invidiousness when it is remembered that true merit is not born but acquired? Ours is a field so large and so varied that we all can find an appropriate place in which to make ourselves useful, and with diligence and perseverance, we may achieve honor and distinction be it with or without title. Attributes of pre-eminence are not always required to command consideration and respect, for the truth of which I refer to a large class of physicians who, by choice or otherwise, follow the more unassuming walks in our profession, but yet fill their places honorably to themselves and usefully to the community. Remember the maxim; "Act well your part therein the honor lies." But whatever the causes of the stagnation of this once progressive Association may be, they cannot outweigh the necessity of its continuance and resuscitation to its former sphere of activity. Organizations, such as this, offer so many advantages for the attainment of that knowledge which qualifies us for the duties we are daily called upon to perform, that to particularize, would require more time and space than I intend to devote to it. A single intellect, with exceptions, will never accomplish that which a number of intellects united are able to accomplish. The advantage of interchange and comparison of views and ideas, besides being reciprocally instructive, teaches a proper estimate of one's self. There is danger for him who keeps himself aloof or isolated, and consequently without the opportunity to compare his views, or measure his attainments with those of his colleagues, that he may become either self sufficient or disheartened, as by nature inclined, but let him join with others in discussions, such as this Association offers, and he will soon find, if disposed to be vain, that others have ideas which he is forced to respect, and on the other hand, if inclined to be disheartened, he finds encouragement in that he possesses knowledge that entitles him to a hearing. The most valuable, however, of the opportunities and advantages which belong to this and similar institutions, is that it offers the stimulus, the incentive to emulate, to excel.

Socially, it brings its members into closer proximity. They learn to regard each other and a mutual feeling of friendship is thereby engendered, having many enjoyable hours in store.

This Association is particularly entitled to the support of the younger members of the profession. An opportunity is here offered them to reap the benefit of mature judgment and long experience pertaining to a multitude of medical subjects. They will never have occasion to regret having attended any of its meetings, for they are certain, each time, to carry away with them one or more ideas that will be of advantage to them at one time or another in the practice of their profession. Besides their every day experience qualifies them to give some equivalent for what they receive, thereby extending their sphere of usefulness.

And here let me say that it is not absolutely necessary, nor is it required of them, to give their experience on subjects of no less importance than a grand operation, as for example ovariotomy, lithotomy or the ligation of the artery in a case of axilary or carotid aneurism; on the contrary, communications, verbal or written, reports on subjects of every day practice, are of equal, if not more value to the members, the greater number of whom are general practitioners, than the details of a grand operation.

But I need no longer dilate on the advantages and opportunities of this Association since the subject embraces facts familiar to you all, and if such facts as I have mentioned, and others which will suggest themselves to the members of the profession, are seriously considered, no fears need be entertained as to the permanency and continued high character of this institution.

In regard to the material affairs of the Association, I must not forget to mention that it has accepted the College Faculty's generous offer of the use of the room we now occupy. The room is well adapted to our purpose and the terms are very acceptable; time of lease, during good behaviour; consideration, our company. As regards the finances, I have no doubt the treasurer will give you a satisfactory report of the balances in or out of his hands.

It would, perhaps, have been more appropriate for me to have addressed you on some more strictly medical subject, but my faith in my ability to contribute anything new or worthy to the common stock of medical knowledge, failed me, and I was constrained to offer you the few remarks I have made, in the hope and with the motive to contribute my mite, towards awakening the Association to new life and vigor.

The Association then proceeded to the election of officers for the ensuing year, with the following result: For President Dr. James P. White, Vice President, Dr. William Gould, Secretary, Dr. E. R. Barnes, Treasurer Dr. J. J. Walsh, Librarian Dr. P. H. Strong.

Dr. White not being present, the Vice President elect, Dr. Gould was, upon motion of Dr. Rochester, conducted to the chair.

Dr. MINER moved that are solution of thanks to the retiring officers be adopted. In presenting this motion, he said that he did it not as a matter of form, but as a recognition on the part of members of the labor these officers had to perform. All had faithfully discharged their duties. The work of the Sectetary required much time and labor, and it should be the duty of members to lighten it as much as possible. Upon the accuracy and intelligence with which the reports of proceedings were made depended the standing of the Society.

The motion of Dr. Miner was carried unanimously.

Dr. HAUENSTEIN requested Dr. Brush to present a report of a case of much interest, of which he, Dr. H., had had charge for a considerable time. In response Dr. BRUSH then read the following paper.

By invitation of the attending physician, Dr. Hauenstein, I present the following report of an amputation at the Hip Joint, which contains some points of interest, as they embody two surgical procedures, which are somewhat novel in character, and have never, I believe, been put in practice previously in this city.

J. W., a German shoemaker, aged 25, has had disease of the right femur involving the knee-joint, since the age of six years.

I can trace no history of traumatic origin, neither does there appear to be any constitutional taint in himself or parents, both of whom are living.

The disease first commenced in the vicinity of the joint by the formation of a large abscess. With the exception of a slight lameness, locomotion was not interfered with until the age of thirteen, when upon recovering from an illness of four months duration, the nature of which I am unable to ascertain, although probably connected with the disease of the bone, the leg was found to be immovably flexed at a right angle upon the thigh.

This was the condition of the patient when first seen by Dr. Hauenstein some ten or twelve months since when he was called to attend him for small-pox. There were several sinuses upon the outer aspect of the limb, from which pus and, at times, spiculæ of bone were discharged. Nothing at that time was done to alleviate the condition of the limb, the patient having consulted several physicians, and being satisfied that nothing could be done for him short of amputating the leg.

About six weeks ago, Dr. Hauenstein was again invited to see the patient, who was suffering with a mild febrile attack, due, probably to the formation of abscesses, two of which were opened. He had partially recovered from this, when upon the 19th or 20th of March, in turning or moving very slightly in bed, he felt something "give way" in his limb, which was accompanied by a "snap" audible in the adjoining room. Upon visiting the patient, the Dr. found evidences of a fracture of the femur about three inches above the knee-joint. Abscesses formed about the seat of the fracture, which, upon being opened, discharged a large amount of offensive pus.

On March 25th, Dr. Miner was called in consultation, and after explaining to the patient the nature of the case, and the risks of the procedure, it was decided to amputate at the junction of the upper and middle thirds of the thigh.

Upon taking into consideration the anæmic and exhausted condition of the patient, Dr. Miner dicided to adopt the plan which was suggested, or rather revived by Prof. Esmarch, of Kiel, at the Surgical Congress of 1873, held in Berlin. This plan, which is but a modification of a method as old almost as Surgery itself, consists in winding an elastic bandage, commencing at the toes tightly around the limb, extending it as high up as the groin. At the upper margin of this bandage a rubber rope is drawn tightly around the limb some three or four times and secured by hooks. The bandage is then removed and the limb is ready for the operation. The case under consideration was an unfavorable one upon which to test this method, owing to the flexed condition of the limb the presence of pus in the tissues and the consequent danger of forcing it into the circulation.

March 26th. After being brought thoroughly under the influence of Ether, the patient was placed upon a table and the appatus applied, as has already been described. Upon removal of the bandage, the limb presented a blanched condition truly remarkable. Dr. Miner amputated in the usual manner, by the flap method. Upon reaching the bone, its condition was not satisfactory, and the flaps were dissected still further up until apparently healthy bone was reached.

Upon making section of the bone, however, pus was seen a exude from the medullary canal, and the only alternative was to remove it at the hip-joint.

Up to this time, sufficient blood had not been lost to stain an ordinary napkin, the tissues presented a bloodless appearance, unlike anything which I have ever observed, and the different structures could be plainly distinguished. The arteries were secured, and the rubber rope removed. The incision was then extended from the outer angle of the wound, parallel with the bone to the upper border of the trochanter major. With the exception of the attachment of the muscles to the linea aspera, the tissues were easily separated from the bone, and the head removed from the acetabulum. Thus making an amputation at the Hip-Joint, after the method described by Prof. Theo. A. McGraw, of Detroit, at the meeting of tne Michigan State Medical Society, in June 1872.

During the whole operation no blood was lost except from the incision made to remove the head of the bone, and less than usually attends the amputation of the leg or forearm, there being in fact, no active hemorrhage at all from vessels of any considerable size. The patient was ordered Morphia, gr. one-sixth every six hours, together with brandy punch, beef tea, etc., etc.

At the request of Dr. Hauenstein, I assumed charge of the patient, and present the following summary of his condition since the operation.

Pulse previous to the operation,

| | | | |
|---|---|---|---|
| March 26th, - - - | 120, | | |
| After operation, | 132, full. | | |
| " 27th, 9.30 A. M., - | 112, | | |
| " 28th, 9.30 " - - | 120, after dressing. | Temp. | $101\frac{1}{2}$ F. |
| " 29th, 9.30 " - | 116, | " | 101 " |
| " 30th, 10 " - - | 112, | " | $102\frac{1}{2}$ " |

The rise in temperature was probably due to obstruction to the free exit of the discharge from the wound. Ordered Quinia gr. iij three times daily. As the pain was not severe to take Morphia less frequently.

| | | | | |
|---|---|---|---|---|
| March 31st, | 10. A. M., | Pulse 110. | Temperature | 101. |
| April 1st, | 10.30, | " 108. | " | $101\frac{1}{2}$. |
| " 2d, | 10.30, | " 108, | " | $101\frac{1}{2}$. |
| " 3d, | 9.30, | " 104, renewed adhesive straps. | | |
| " 4th, | 10.30, | " 106, | Temp. | 101. |
| " 5th, | 10.30, | " 108, | | |
| " 6th, | 12 M, | " 106, | | |
| " 7th, | 10.30, | " 104, | | 100. |

Appetite good, stump looking well, and pain but slight.

This case presents several points of interest which deserve a passing notice.

The origin of the disease is of so early a date that it is impossible to arrive at any definite conclusions respecting its cause. What the condition of the bone really is, I leave for those more experience in such matters than myself, to determine. It will be observed that at the point of fracture, the bone presents the appearance of caries, this is also observable in the region of the condyles. The Patella is seen to be united to the Femur by bony deposit. The Tibia presents a healthy appearance; its articulating surface however, is smaller than usual, and the spine less prominent. The head of the Fibula is of natural size, but its shaft is remarkably small, owing doubtless, to long disuse, the patient having used crutches for thirteen years, during which time the weight of the body was never borne upon the limb.

When we come to examine the head and neck of the femur a a curious malformation is noticeable. The upper portion of the head is flattened, but apparently healthy. The spontaneous fracture of the femur is interesting as an instance a of somewhat rare accident; it will be seen however, that the bone had reached a condition of disease, in which, but little force was necessary to produce the fracture.

The method proposed by Esmarch, and here put in practice, was advanced as a new procedure in Surgery, but all familliar with surgical literature and practice, will recognize it as an old idea newly introduced and modified.

The rarity of recovery after amputation at the Hip-Joint, gives additional interest to this case.

Dr. Otis gives the following statistics of this operation in Civil Practice.

| | |
|---|---|
| In France, 8 successful, | In Germany, 7 successful. |
| " Gr. Britain, 16 " | " America, 15 " |
| In France, 15 unsuccessful. | In Germany, 6 unsuccessful. |
| " England, 31 " | " Poland, 4 " |
| " America, 9 " | |

Total number 46 successful. Failures 65.

He gives in Military Practice both American and Foreign 166 cases, with 142 deaths; 16 recoveries and 3 doubtful.

Dr. MINER said that Esmarch's suggestion was not new in principal. Thirteen years ago, at the Buffalo General Hospital, with Drs. Eastman and Wilcox, he had made use of the same procedure, using a roller bandage and a tourniquet, instead of the rubber band and tubing; the method and purpose being the same, but the material used, different. The elastic band more fully empties the vessels than the roller-bandage, as each successive turn expels the blood without diminishing the pressure of those below. Our former townsman, L. Danforth, now deceased, obtained a piece of rubber in the form of a band and presented it to this Association, with the recommendation, that it would be of service to soldiers for the purpose of arresting hemorrhage. In presenting this band, Dr. Miner had explained its advantages as a tourniquet and bandage. See Vol. 1, No. 5, Buffalo Medical and Surgical Journal, Dec., 1861. Dr. M. thought the plain rubber band better than the elastic webbing now sold, for the purpose under consideration. In making these statements he did not wish to detract from the credit due to Esmarch. His merit was great in having reintroduced this procedure, and authoritatively called the attention of the profession to its advantages. By this method we may amputate and perform other operations without loss of blood. It is astonishing how perfectly this object is attained. There are many operations in which this procedure is inapplicable. There is danger sometimes of forcing deleterious substances upwards into the tissues, or into the circulation. Generally, if there are collections of pus, as in the case just reported, this procedure is not contra-indicated, when the pus is contained in cavities or sinuses, not favorable to absorption. In this case there was very little shock from the operation, and the system reacted readily. As will be seen from the statistics presented by Dr. Brush, the proportion of recoveries after amputation at the hip-joint, is very small. From particular localities, and under certain conditions, as in war, the reports are much less favorable. All the cases reported from Boston were unsuccessful, while, very few recoveries are recorded in military practice. It had been recommended by Woodbury of Philadelphia, to introduce the hand into the rectum and compress the illiac artery or even the aorta. Dr. Miner had never amputated at the hip-joint before. In the case reported by Dr. Brush, he attributed the favorable result more to the method employed of amputating low down, and then removing the bone, than to the use of the bandage. The nearer the point of section is to the trunk, the greater the danger. The length of stump secured by the method referred to, is of little or no service in adapting an artificial leg, but it permits the division of the limb at a distance from the trunk, it relieves shock, ensures less loss of blood, and a division of less muscular tissue. Would never amputate at the hip or shoulder joint by the old method if it were possible to employ the other. He had several times amputated successfully at the shoulder-joint in this manner, in unfavorable cases, as also had Dr. Barnes, in one case at the General Hospital.

The prevailing diseases reported, were sore throat, often accompanied by swelling of the parotid glands, sometimes by a discharge from the ear, typhoid fever very prevalent, scarlet fever, measles, diarrhœa and articular rheumatism.

Miscellaneous business being in order, the question was raised as to securing copies of the transactions of the State Medical Society but no definite action was taken.

On motion the Treasurer was authorized to pay Dr. Rochester, one hundred dollars, for rent of rooms for the Association, for the two past years.

On motion, adjourned.

